# Early Estimation of the Reproduction Number in the Presence of Imported Cases: Pandemic Influenza H1N1-2009 in New Zealand

**DOI:** 10.1371/journal.pone.0017835

**Published:** 2011-05-26

**Authors:** Michael George Roberts, Hiroshi Nishiura

**Affiliations:** 1 Centre for Mathematical Biology, Institute of Information and Mathematical Sciences, and New Zealand Institute for Advanced Study, Massey University, Auckland, New Zealand; 2 PRESTO, Japan Science and Technology Agency, Saitama, Japan; 3 Theoretical Epidemiology, University of Utrecht, Utrecht, The Netherlands; University of Oxford, Viet Nam

## Abstract

We analyse data from the early epidemic of H1N1-2009 in New Zealand, and estimate the reproduction number 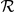. We employ a renewal process which accounts for imported cases, illustrate some technical pitfalls, and propose a novel estimation method to address these pitfalls. Explicitly accounting for the infection-age distribution of imported cases and for the delay in transmission dynamics due to international travel, 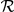 was estimated to be 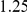 (95% confidence interval: 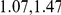). Hence we show that a previous study, which did not account for these factors, overestimated 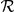. Our approach also permitted us to examine the infection-age at which secondary transmission occurs as a function of calendar time, demonstrating the downward bias during the beginning of the epidemic. These technical issues may compromise the usefulness of a well-known estimator of 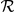 - the inverse of the moment-generating function of the generation time given the intrinsic growth rate. Explicit modelling of the infection-age distribution among imported cases and the examination of the time dependency of the generation time play key roles in avoiding a biased estimate of 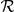, especially when one only has data covering a short time interval during the early growth phase of the epidemic.

## Introduction

Influenza A (H1N1-2009) emerged in 2009 [1]. The ensuing pandemic precipitated an international effort to quantify epidemiological parameters, as a necessary first step to assessing its potential impact [2]. Among epidemiological quantities, the most commonly used determinant of the transmission potential has been the basic reproduction number 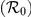, defined as the expected number of secondary cases arising from a typical primary case throughout its entire course of infection in a fully susceptible population. The value of 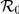 is a primary determinant of the size of an epidemic and the effort required to contain it [3], [4]. Given that 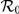 has been theoretically defined for a fully susceptible population, we (in common with other authors, e.g. [5], [6]) refer to the reproduction number 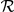, which we estimate from the initial growth phase of the epidemic [7], [8]. Such an estimate can aid public health decision-making in real-time during the course of a pandemic [9], [10].

The emergence of H1N1-2009 was first detected in North America in March 2009, and initial estimates of its reproduction number, ranging from 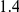 to 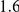 [6], and from 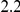 to 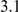 [5], were published in May of the same year, and derived from Mexican data. Because the emergence in Mexico was at the same time as the beginning of the winter influenza season for Southern Hemisphere countries, it was important to determine if there was a higher transmission potential under winter conditions. A preliminary study in New Zealand estimated 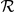 to be in the range 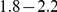 [11], determined from the exponential growth rate of locally transmitted cases and the assumption that the generation time was known, with a mean of 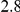 days. Another study in Victoria, Australia, estimated the reproduction number to be in the range 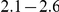 [12]. Later analyses of the same datasets from New Zealand and Australia, which distinguished imported cases from local cases, estimated the instantaneous (effective) reproduction number as a function of time, and the highest estimate appeared to be smaller than those published in the earlier studies (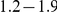 for New Zealand and 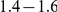 for Victoria) [13], [14]. Because the H1N1-2009 pandemic in these countries (and all the countries other than Mexico) involved repeated introductions of imported cases, it is essential to explicitly account for this aspect in order to appropriately model the transmission dynamics.

Despite the recognition of the role of imported cases in New Zealand, we have yet to clarify the reasons behind the overestimation of 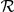 in the above-mentioned study [11], [15]. The purposes of the present study are to illustrate two technical pitfalls in estimating 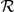 during the early epidemic growth phase, and to offer a novel estimation method for 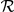 in the presence of imported cases. Because one should know the best method of obtaining an unbiased estimate of 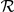 in a similar setting, in order to give appropriate feedback to the public health authorities, we compare different modelling strategies for estimating 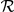 in the presence of imported cases. In the next section, we describe the H1N1-2009 epidemic in New Zealand, and illustrate of the estimation method for 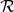 as used in the earlier study. We then explore the underlying reasons for the overestimation of 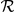. Although a potentially important source of error is heterogeneous mixing (e.g. age-related heterogeneity and other social contact structures), we will not discuss this. Heterogeneous mixing was important in Japan [16], but there was no strong signature of clustering of cases among children during the containment phase in New Zealand. The proportion of children among local confirmed cases by 22 June was as small as 51.4%, and the mean and median ages of local confirmed cases were 22.4 and 19.0 years, respectively. Rather than age-related heterogeneity, we describe two critical factors, one of which is concerned with an explicit modelling approach to imported cases.

In the next section we describe the epidemic in New Zealand, and reexamine the data for the incidence of infection. We then propose a model for the epidemic, based on a renewal process with immigration. The proposed model is used as the basis for a statistical estimation of 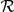, and we conclude with some remarks concerning the infection-age distribution.

## Methods

### H1N1-2009 in New Zealand

**Figure 1:**
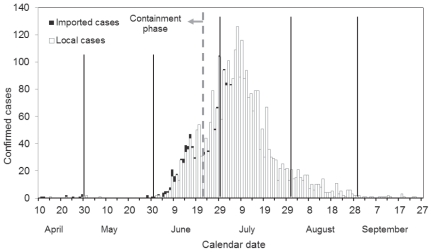
The daily incidence of H1N1-2009 in New Zealand from April to September 2009. Only confirmed cases are shown. White bars represent local cases (i.e. locally transmitted cases without overseas travel), black bars represent imported cases. Vertical solid lines indicate the last calendar date of each month. The vertical dashed line is at June 22, the date on which the control policy switched from a containment to a management phase.

The daily incidence of confirmed cases of H1N1-2009 in New Zealand is shown in Figure 1. The first cases were in a group of students who had visited Mexico and returned on April 25 [11]. The infection was declared notifiable shortly afterwards, and cases were recorded in the *EpiSurv* database. The date of incidence in Figure 1 is assumed to be the earliest date provided on the database, which may be either the date of symptom onset, hospitalisation, death or reporting. Since the data do not offer further information, we hereafter regard the earliest recorded date as the date of infection (see Discussion). As was adopted elsewhere [17], [18], cases with a history of overseas travel within 10 days preceding the onset of illness tend to be defined as imported cases. Since we examine only early epidemic phase without obvious clustering among locally-acquired cases, we assume that no misclassification has occurred in distinguishing between imported and locally-acquired cases.

The initial outbreak declined to extinction in early May and the major epidemic began in June. Until June 22, all reported cases were subject to confirmatory diagnosis and were consistently recorded. On June 22 the health authorities switched the control policy from a *containment* to a *management* phase. During the latter phase not all cases were confirmed, hence the reporting coverage must have been incomplete. The last confirmed case in New Zealand in 2009 was recorded in the database on December 29. A total of 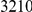 confirmed cases were recorded. Because we estimate 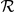 from the early epidemic growth phase (when the cases should ideally be recorded consistently over time), we limit our analyses to the containment phase before June 22.

Let 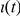 be the incidence (i.e. the number of new cases) at calendar time 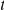. During the early growth phase, each primary case generates on average 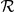 secondary cases. The relative frequency of secondary transmission with respect to the time since infection of a primary case is denoted by 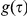, which is referred to as the generation time (and 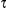 is referred to as infection-age). The expected number of new cases 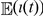 in the absence of imported cases is written (e.g. [3], [19]–[21])
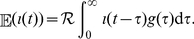
(1)When the incidence grows exponentially with growth rate 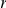, we have 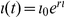 where 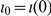, a constant. Replacing 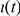 on the both sides of (1):
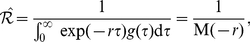
(2)where 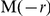 is the moment-generating function of the generation time, given the intrinsic growth rate 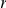 [8]. Hence 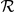 can be estimated, given an estimate of 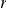 and if the generation time distribution 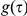 is assumed to be known [7], [8].

We should not ignore demographic stochasticity during the early growth phase of an outbreak, hence the following pure birth process is useful when estimating 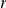 [22], [23]. Let 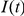 be the cumulative incidence at time 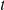. Then
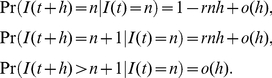
(3)For the analytic solution of equations (3) see [24]. Given our observations of the cumulative number of cases, we have
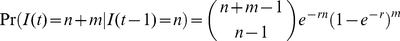
(4)which can be used as a conditional likelihood function to estimate 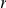.

**Figure 2:**
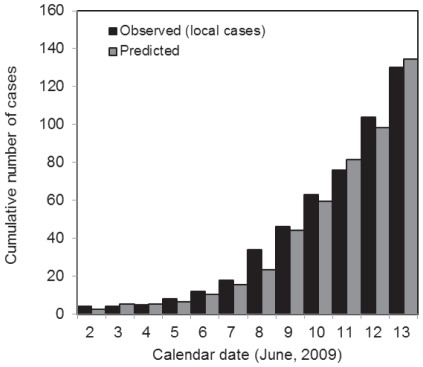
Observed (black) and predicted (grey) cumulative numbers of confirmed locally transmitted cases. Predicted values represent conditional expectations given by 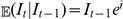 where 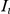 is the cumulative number of cases at day 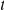, and 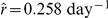 is the maximum likelihood estimate of the growth rate.

The observed and predicted cumulative numbers of local confirmed cases are shown in Figure 2. Although the earliest dates of incidence in Figure 1 have been refined and are different from those analysed in an earlier study [11], the estimated growth rate from 2–13 June is 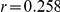 day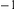 (95% confidence interval (CI): 0.219, 0.302), which is consistent with the estimate in [11]. The mean 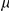 and variance 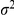 of the generation time have been estimated from contact tracing in the Netherlands to be 2.70 days and 1.21 days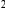, respectively [25]. Assuming that the generation time follows a gamma distribution, the estimator of 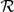 based on equation (2) is 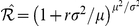, leading to 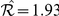 (95% CI: 1.76, 2.15). This is high compared with published estimates from other countries (e.g. [5], [6], [12], [16], [26], [27]), and is likely to be an overestimate.

### A general renewal process with imported cases

When analysing data for the initial growth of an epidemic it is important to account for imported cases correctly. In equation (2) a stochastic pure birth process was fitted to local cases alone. In discarding imported cases, we correctly removed cases that would otherwise be counted as secondary cases, but at the same time removed some primary cases. This could artificially elevate the estimate of 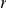, and thus 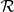. In the presence of imported cases with incidence 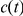 at time 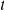, the renewal process (1) could read
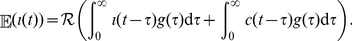
(5)Equation (5) is a general form of the age-dependent branching process with immigration [28]. Although equation (5) does not account for different infection-age distribution among imported cases (as compared to local cases), models of this type have been applied to data sets for H1N1-2009 in several published studies [13], [14], [26] If we have 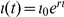, the estimator of 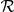 is
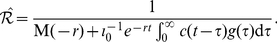
(6)The denominator on the right-hand side includes the imported cases, 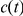, and hence a solution requires an approximation to 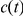. At the very least, equation (6) highlights that the estimate of 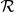 based on equation (2) results in an overestimate in the presence of imported cases.

Equation (5) requires further modification to capture the underlying dynamics of the epidemic. Before being diagnosed in New Zealand, imported cases were infected overseas, hence there was a time-lag from their infection to their involvement in local transmission. To approximate this, we introduce a constant delay in the involvement of imported cases, i.e.
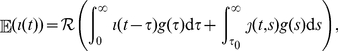
(7)where 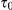 represents the time taken from infection to importation (for example the time taken for an international flight). The shortest connecting flight from Mexico City to Auckland is 20 hours 30 minutes, hence we assume that 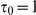 day. The importance in capturing this delay has been emphasised elsewhere [15]. We have ignored possible transmission during transit. In reality, the infection-age distribution among imported cases is influenced by the transmission dynamics at the origin of importation. However the second integral contains the term 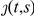, the number of new imported cases arriving at time 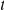 and infection-age 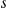 (instead of 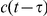). The times of infection among imported cases are seldom known, hence we postulate an epidemic process at the origin of importation. First, assume that 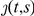 may be expressed by the convolution
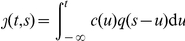
where 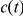 corresponds to the observed counts of new imported cases at time 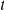, and 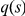 is the density function of the infection-age of imported cases. Second, assume the incidence of infection at the origin of importation to be approximated by an exponential with the same growth rate 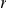 as that in New Zealand. That is, we assume that the epidemic is in an early phase at the origin of importation and the growth of cases is sufficiently approximated by deterministic exponential growth. The density function of the infection-age of imported cases, 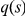 is then given by
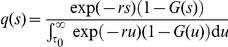
for 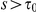 [29] and 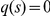 otherwise. It should be noted that dynamics other than exponential growth would require us to examine additional data (e.g. epidemic data at the origin of importation), but our assumption permits us to account for the infection-age of imported cases by using local epidemic data only (i.e. the data set in New Zealand). Consequently, the time- and age-dependent number of imported cases is modelled as
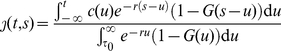
(8)in equation (7). The growth rate 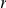 in the right-hand side of (8) can be replaced by 
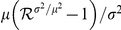
 as in the estimator of 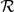 described above.

In summary, we have devised a modelling approach to early epidemic processes with imported cases that accounts for two issues. The first is a constant delay (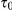) in transmission dynamics involving imported cases, which corresponds to the time in transit. The second is a distributed delay. The imported cases are likely to have been infected shortly before departure, but their infection-age distribution should take account of transmission dynamics at their origin. There are other factors that could add further detail: for example the relative contribution of imported cases to secondary transmission in comparison with local cases (see [15] for a description), and heterogeneous mixing. However, we have insufficient data to account for these. It should be noted that the time-dependency of imported cases in the proposed model (7) does not lead to an exponential growth of local cases. Simpler age-dependent branching process models with immigration have been examined elsewhere to find the analytical solutions to describe the growth of local cases [30], [31].

### Statistical estimation of 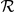


**Figure 3:**
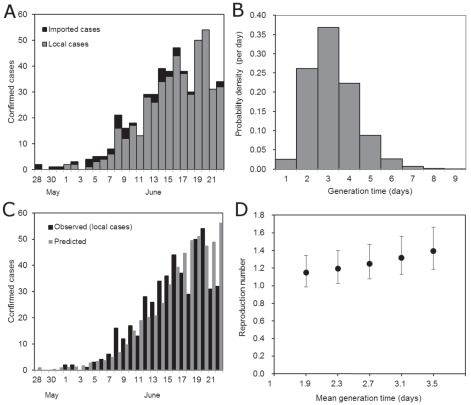
Transmission dynamics of H1N1-2009 in New Zealand. A: Observed daily incidence of imported (black) and local (grey) cases from 28 May to 22 June 2009. We examine only confirmed cases during the containment phase. B: Discretised distribution of the generation time. Mean and variance are assumed to be 2.70 days and 1.21 days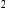, respectively. C: Observed (black) and predicted (grey) numbers of local confirmed cases. Predicted values represent conditional expectations derived from our proposed model, which includes adopting a negative binomial offspring distribution. D: Sensitivity of the estimated reproduction number to the mean generation time, over the range 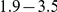 days. Whiskers extend to the upper and lower 95% confidence intervals based on the profile likelihood.

We now estimate 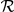 using the modelling approaches described above. Figure 3A shows the daily incidence of confirmed imported (black) and local (grey) cases from 28 May to 22 June 2009. Since we failed to jointly estimate 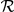 and the generation time distribution (see below), we assume that the generation time distribution 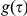 is known, with the mean 2.70 days and the variance 1.21 days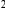 [25], but we examine the sensitivity of 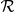 to the mean generation time. While 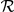 may vary by location, over time and according to the level of public health interventions, we generally expect the generation time to be consistent between locations, unless extrinsic measures significantly influence epidemiological patterns of transmission [32]. Since the observed data are provided as daily reports, we discretize the distribution,
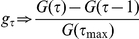
for 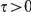, with 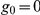, and where 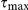 is the longest infection-age causing secondary transmission. In the following we set 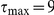, because the frequency of secondary transmission after infection-age 9 days is negligible.

We examine three different models to illustrate the impact of underlying assumptions with regard to imported cases on the estimate of 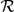, and to determine the best modelling strategy. Let 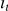 and 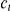 be the incidence of local and imported cases on day 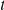, respectively. We denote the history of both series of cases up to day 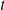 by 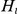. The first model we examine is the renewal equation with imported cases, but without an adjustment of infection-age distribution and without a delay. Given 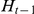, the conditional expected incidence of local cases on day 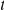 is
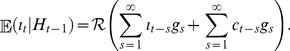
(9)The second model accounts for a constant delay in imported cases, but without adjustment for their infection-age distribution, i.e.
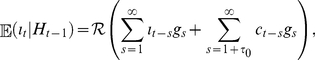
(10)where 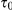 is the time taken for transit, assumed to be one day. In the third model, we incorporate the adjustment of infection-age distribution and a constant delay in the transmission dynamics:
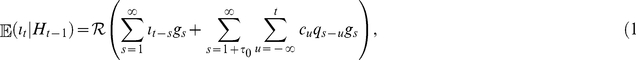
(11)where
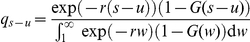
and 
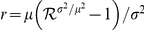
.

We examine two conditional likelihood functions for the estimation of 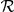. First, if we regard 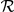 as a (deterministic) parameter and ignore individual heterogeneity in the number of secondary transmissions, then the infection process is Poisson [3]. Assuming that the discrete generation time follows a multinomial distribution, a thinned Poisson is obtained [33] which is known to be useful for the joint estimation of 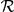 and 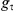 [26], [33]:
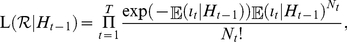
(12)where 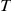 is the last date of observation (equivalent to 22 June 2009) and 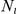 represents the observed number of local cases on day 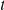. As an alternative, we incorporate a gamma-distributed individual heterogeneity for the infection process, which results in a negative binomial distribution [6], [34]:
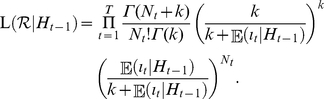
(13)The dispersion parameter 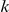 has to be jointly estimated when employing equation (13). The Poisson distribution is obtained as 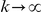, and the logarithmic series distribution is obtained as 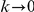. A maximum likelihood estimate of 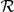 (and additionally, 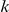 for the negative binomial likelihood distribution when applicable) is obtained by minimizing the negative logarithm of either (12) or (13), and the 95% CI is derived from the profile likelihood. To compare model fit we employ Akaike's Information Criterion, 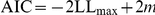, where 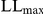 is the maximum value of the loglikelihood function and 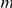 is the number of parameters estimated.

### Assessment of the infection-age distribution

In equations (9–11), the right-hand side inside parenthesis (i.e. other than the factor 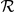) may be interpreted as the expected number of cases who have a potential to cause transmission at time 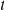 (We refer to these as primary cases). For example, using the best-fit model (11), the expected number of primary cases is
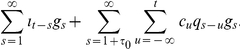
The mean infection-age at which secondary transmission has occurred is
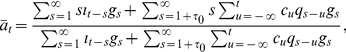
(14)and the variance is
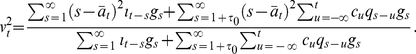
(15)


The time required for the generation time to converge to a stable distribution has attracted the recent attention of epidemic modellers [35], but this has been preceded by discussions in the mathematical demography literature for more than 30 years. The population entropy, proposed by Lloyd Demetrius, is defined by
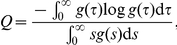
and has been shown to measure the rate of convergence of a population to a stable age distribution [36]. Further theoretical accounts of 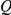, and insights into its interpretation employing a Leslie model, are described in [37].

## Results

### Estimates of the reproduction number

The maximum likelihood estimates of 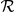 ranged from 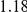 to 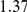, see Table 1. Where different offspring distributions were used with the same type of model, the negative binomial distribution resulted in a better fit than the Poisson distribution (lower AIC). In addition, the negative binomial distribution always led to a greater 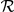 than the Poisson distribution, but with wider uncertainty bounds reflecting its fatter tail. When different models were compared, the model that accounted for the infection-age distribution and a constant delay for imported cases (AD) was the best fit, and resulted in the estimate 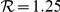 (95% CI: 1.07, 1.47). Differences in the estimates of 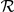 were very small between models with and without a constant delay for imported cases (RP & RD). This is because 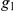 of the assumed generation time distribution is small, but if the generation time were shorter than assumed, its influence would be greater [15].

**Table 1:** Comparison of parameter estimates and model fit.

Model^1^	Offspring distribution^2^	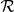 (95% CI)^3^	AIC^4^	Dispersion parameter^5^
RP	P	1.22 (1.11, 1.33)	168.9	
RP	NB	1.36 (1.13, 1.66)	149.9	10.2 (3.8, 30.1)
RD	P	1.22 (1.11, 1.33)	169.6	
RD	NB	1.37 (1.14, 1.68)	150.1	10.0 (3.7, 29.1)
AD	P	1.18 (1.08, 1.28)	157.2	
AD	NB	1.25 (1.07, 1.47)	144.3	14.4 (5.1, 48.2)

1 RP: renewal process, equation (9); RD: RP plus a constant delay in imported cases, equation (10); AD: RD plus statistical adjustment of infection-age distribution among imported cases, equation (11).

2 P: Poisson distribution, equation (12); NB: negative binomial distribution, equation (13).

3 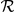: reproduction number, CI: confidence intervals derived from profile likelihood.

4 AIC: Akaike Information Criterion.

5 Dispersion parameter of negative binomial distribution.

In Figure 3C the observed and expected (based on the best-fit model) numbers of locally transmitted confirmed cases are compared as a function of time. In Figure 3D the sensitivity of 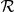 to different mean generation times, ranging from 1.9 days to 4.0 days (assumed maximum), is examined. As in previous studies [7], [8], as the mean generation time increases the estimate of 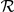 also increases (ranging from 1.15–1.39). This illustrates the importance of having a reliable estimate of the generation time distribution if one is to obtain a precise estimate of 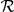.

In addition to the results shown in Table 1, we attempted to jointly estimate 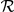 and the generation time distribution using a Poisson-distributed likelihood function. Employing a model with a one day delay for importation, and limiting the maximum generation time to 4 days, we obtained 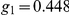, 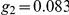, 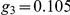 and 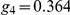. Thus, the mean of the jointly estimated generation time was 2.38 days. Attempts to estimate with a greater maximum generation time did not result in successful convergence. We know of no explanation for the implied bimodal distribution, so regard this as a failure to implement a joint estimation. We discuss this outcome in the next section.

### Infection-age at which secondary transmission occurs

**Figure 4:**
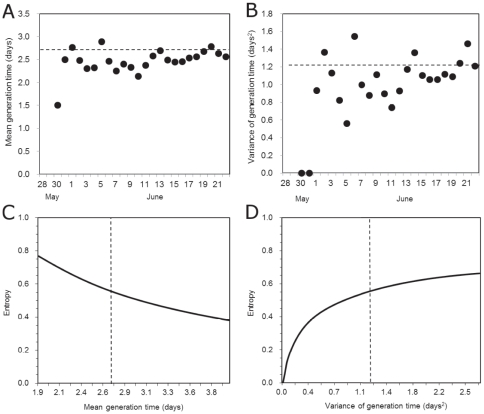
Assessment of the distribution of generation time. A: The mean generation time as a function of calendar time. B: The variance of the generation time as a function of time. C: Sensitivity of population entropy to mean generation time. D: Sensitivity of population entropy to the variance of the generation time. The horizontal dashed line in A, and the vertical dashed line in C, represents the assumed mean generation time, 2.70 days; which is fixed in B and D. The horizontal dashed line in B, and the vertical dashed line in D, represents the assumed variance, 1.21 days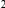; which is fixed in A and C.

In Figure 4A the mean generation time (i.e. the mean infection-age at which secondary transmission occurs) is shown as a function of time, as derived from equation (14). Initially, the mean infection-age of secondary transmission is small, and is shorter than the assumed mean generation time, 2.70 days. As the epidemic progresses, the mean generation time increases and converges to the assumed mean. Despite its convergence to 2.70 days, the mean generation time tends to be short during the first 2 weeks of the epidemic. In Figure 4B the variance of the generation time is shown to fluctuate as a function of time (from equation (15)), before converging to the assumed variance.

The assessment of the time-dependent generation time distirbution plays a key role in interpreting the reason behind the overestimation of 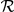 when employing the exponential growth rate 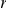 based on a pure birth process (4). It must be noted that the well-known estimator 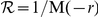 depends on the assumption that the infection-age distribution is stable. If not stable, the direct application of the estimator could yield a biased estimate of 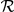. Even provided that 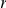 is estimated to be as large as 0.258 day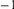 during the initial phase of the epidemic, the estimate reflects transmission that occurrs at earlier infection-ages than the mean generation time. The illustrated time-dependency of the generation time distribution also partly explains the failure of the joint estimation of 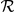 and the generation time reported above. Given that the majority of observed transmission events take place at early infection-ages, and given that the variance has also fluctuated, a precise estimate of the generation time distribution is not possible. In fact, the jointly estimated generation time would be shorter than an unbiased estimate of the generation time. A successful joint estimation would require a longer time series of data than we examined. In addition, a recent study has shown that the joint estimation involves several technical difficulties during the early exponential growth phase of an epidemic, especially in the presence of heterogeneous transmission [38].

In Figure 4C the sensitivity of 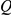 to the mean generation time is examined. It is evident that the time taken for convergence is longer when the mean generation time is longer. In addition, it is important to examine the influence of the variance of the generation time on 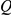 (Figure 4D). If the variance were zero (i.e. for a delta function), the infection-age distribution would not converge to a stable distribution. As the variance increases, convergence improves. Since the reporting interval for influenza (i.e. daily data) is similar to the mean generation time, the time taken for convergence is less likely to be a significant problem than it would be for slower diseases (e.g. HIV/AIDS). Nevertheless, this issue cannot be ignored when we estimate 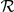 from a dataset covering a short period of time during the early growth phase. Indeed, the time-dependent infection-age distribution is a plausible explanation for an overestimation using the growth rate 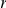. In addition to the issue of precise estimation of 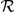 from early epidemic growth data, this highlights the critical importance of quantifying the generation time distribution, and especially its variance, if we are to understand the underlying epidemic dynamics.

## Discussion

We have estimated the reproduction number 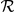 for H1N1-2009 in New Zealand, by reanalysing the early epidemic growth data. We explored two modelling issues: taking account of imported cases; and the infection-age distribution at which secondary transmission occurs during the early growth phase of the epidemic. We believe these provide at least part of the underlying reasons for a previous overestimate of 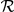 [11]. Explicitly accounting for the infection-age distribution of imported case, and the delay due to transit, 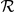 was estimated to be 1.25 (95% CI: 1.07, 1.47). Despite wide uncertainty, the upper 95% CI is smaller than the lower 95% CI of the published preliminary estimate [11]. Moreover, our modelling approach permitted us to examine the generation time as a function of calendar time, demonstrating that generation time is biased downwards during the beginning of the epidemic. Both points illustrate important technical pitfalls in the use of the exponential growth rate 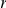 and the estimator 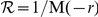 for early growth data. To avoid a biased estimate of 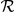, we propose investigation of both of these issues, especially when one has to measure 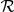 from data collected over a short period of time during the early growth phase.

We have shown that explicitly accounting for imported cases would be a key factor in avoiding an overestimation of 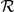. We have also emphasised the importance of addressing the infection-age distribution for imported cases, which will be different to that for local cases. When modelling transmission from imported cases, one should account for the time-lag from infection to importation, and account for the transmission dynamics at the origin of importation. The former can be approximated by a fixed delay, the average time required for international travel. The latter requires an assumption concerning the transmission dynamics at the origin. In addition, the use of a negative binomial offspring distribution was favoured for the three models we examined. Demographic stochasticity during the early growth phase is not negligible, and it appears that the stochastic early epidemic process in New Zealand was better captured by the negative binomial distribution than the Poisson distribution, indicating the presence of individual heterogeneity in the transmission process.

One implication of the proposed model is that the generation time was yet to converge to a stable distribution in New Zealand at June 22, 2009. In particular, the mean infection-age at which secondary transmission occurred appeared to be short, partially explaining the reason for the overestimation of 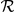. It must be remembered that the estimator 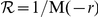 is based on the assumption that the generation time is stable, and this is frequently not the case early in the epidemic. One should then employ a renewal process (equations (1) and (11) in the absence and presence of imported cases, respectively) and estimate 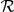 as a parameter. The population entropy 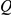 indicates the rate of convergence to a stable distribution.

Four limitations of this analysis should be noted. First, our estimate of 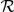 is based on the daily incidences of confirmed cases, which are recorded when an infection is classified as *notifiable*. As with any data set there could be issues with classification and interpretation, but these are the best items of information available at the time. In particular, the earliest date recorded has been taken as a proxy for the date of infection for locally transmitted cases. Further in-depth investigation of each case (e.g. taking account of the incidence and reporting delay) could potentially produce a more accurate data set, but our objective is to produce an estimate based on the information to hand. Second, although we recognise the crucial role of the generation time distribution, we have based our distribution 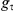 on the published result of a contact tracing exercise [25]. Despite the existing method for estimating 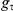 in real time [33], we have yet to invent a method for its unbiased estimation [38]–[40]. Third, we have ignored heterogeneity (other than infection-age) and adopted the homogeneous mixing assumption. As has been discussed elsewhere [6], [12], [22], [27], age-related heterogeneity is likely to provide additional insights into the transmission dynamics, and estimation of the relative contribution of imported cases to secondary transmission (compared with local cases) should be possible through examining additional epidemiological information. Fourth, although unlikely to vary the results of the present study (because the flow of cases can be assumed to be unidirectional from North America to New Zealand), we focused on the mobility of primary cases and did not discuss that of secondary cases. Where emigration would influence the growth estimate of cases (e.g. in Mexico or for a metapopulation model), models with bidirectional mobility would be called for.

In conclusion, the early epidemic data in New Zealand did not suggest that the transmission potential of H1N1-2009 was higher than in Northern Hemisphere countries [6], [16], [26], [27]. The present study has highlighted the importance of modelling the transmission dynamics of imported cases and examining the infection-age distribution of primary cases during the early stage of an epidemic, and we believe that these aspects explain some of the reasons for the overestimation of 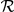 in an earlier study [11]. When it is necessary to obtain an estimate of the transmission potential for a novel emerging disease, we suggest the use of equation (11) with a negative binomial offspring distribution, and equation (14) for the assessment of the mean infection-age of primary cases.
